# Impact of Inflammatory Bowel Disease on Quality of Life and Work Productivity in Patients Under Treatment

**DOI:** 10.7759/cureus.97297

**Published:** 2025-11-19

**Authors:** Nikolaos Kafalis, Dionysios Kogias, Vasileios P Papadopoulos, Ioannis Moschos, Panagiotis Skendros, Konstantinos Ritis, Georgios Kouklakis

**Affiliations:** 1 First Department of Internal Medicine, Democritus University of Thrace, Alexandroupolis, GRC; 2 Gastroenterology-Hepatology Unit, University Hospital of Alexandroupolis, Alexandroupolis, GRC; 3 Laboratory of Anatomy, Department of Medicine, Democritus University of Thrace, Alexandroupolis, GRC; 4 Department of Nursing, International Hellenic University, Thessaloniki, GRC

**Keywords:** crohn’s disease, inflammatory bowel disease, quality of life, ulcerative colitis, work productivity

## Abstract

Background

Inflammatory bowel disease (IBD), encompassing Crohn’s disease (CD) and ulcerative colitis (UC), is a chronic immune-mediated disorder that often emerges in early adulthood, coinciding with peak occupational years. Despite therapeutic progress, IBD continues to impair health-related quality of life (HRQoL) and work capacity. This study aimed to assess its impact on quality of life and work productivity compared with healthy controls.

Methodology

This cross-sectional, observational study included 371 patients with IBD (CD: 59.8%; UC: 40.2%) and 371 age- and gender-matched healthy controls, recruited from two tertiary hospitals. Clinical, endoscopic, and demographic data were collected, including disease duration, treatment regimen, disease activity, and the presence of extraintestinal manifestations. HRQoL was assessed using the EuroQol 5 Dimensions 3 Levels (EQ-5D-3L), and work productivity was assessed using the Work Productivity and Activity Impairment (WPAI) questionnaire. Predictive models were implemented using categorical regression with optimal scaling.

Results

Across all dimensions of the EQ-5D-3L and WPAI, patients with IBD reported greater impairment than healthy controls (p < 0.001). Multivariable models identified physically demanding occupation as the strongest predictor of impaired patient-reported outcomes, with extraintestinal manifestations, age, gender, and disease duration contributing variably across domains (all p < 0.05). CD was independently associated with limitations in usual activities (p = 0.007). Both the Harvey-Bradshaw Index (p < 0.001) and Partial Mayo Score (p < 0.001) correlated negatively with health state and activity impairment.

Conclusions

IBD patients under treatment continue to experience significant impairments in HRQoL and work productivity compared with healthy controls. Physically demanding occupation, extraintestinal manifestations, and CD emerged as key determinants of impaired outcomes in IBD, highlighting the need for integrated management strategies addressing occupational and systemic disease burden.

## Introduction

Inflammatory bowel disease (IBD), comprising Crohn’s disease (CD) and ulcerative colitis (UC), is a chronic, immune-mediated condition that primarily affects the gastrointestinal tract but is also associated with systemic and extraintestinal manifestations (EIMs) [1]. The disease typically presents during early adulthood and persists throughout life, requiring long-term treatment and monitoring. Despite advances in pharmacological therapies, including immunomodulators and biologic agents, a considerable proportion of patients continue to experience fluctuating disease activity, persistent symptoms, or complications that significantly affect daily functioning and psychosocial well-being [1,2].

Beyond its clinical presentation, IBD imposes a substantial burden on health-related quality of life (HRQoL). Patients frequently report limitations in physical functioning, fatigue, pain, social isolation, and emotional distress such as anxiety and depression, even during periods of disease remission [3,4]. These factors collectively contribute to a reduced perception of overall health and well-being. The EuroQol 5 Dimensions 3 Levels (EQ-5D-3L) is a widely validated, standardized instrument used to assess HRQoL across five dimensions, namely, mobility, self-care, usual activities, pain/discomfort, and anxiety/depression, and has been applied extensively in chronic disease populations, including those with IBD [5].

In addition to quality of life, IBD affects work productivity and occupational participation. The unpredictable nature of disease flares, need for frequent medical visits, fatigue, and associated symptoms may lead to absenteeism, reduced performance while at work (presenteeism), and even long-term job loss [3]. The Work Productivity and Activity Impairment (WPAI) questionnaire is a validated tool that quantifies these impairments in both paid and unpaid work [6]. Previous studies have shown that IBD patients incur significant productivity losses compared to the general population, contributing to the economic and societal burden of the disease [7,8].

While the impact of IBD on quality of life and work function is well acknowledged, most data derive from populations with uncontrolled or severe disease [9-11]. Less is known about the residual burden among patients who are actively receiving treatment and engaged in care. Given that modern therapies aim not only to induce remission but also to improve long-term functioning, evaluating the real-world impact of IBD under treatment is essential for guiding holistic care.

The present study aimed to assess the impact of IBD on HRQoL and work productivity in patients under treatment in comparison to age- and gender-matched healthy controls. Using validated tools (EQ-5D-3L and WPAI), we sought to quantify the functional and occupational burden of IBD and identify clinical factors associated with greater impairment.

## Materials and methods

Study design and population

This was a cross-sectional, case-control, observational study designed to compare patients with IBD receiving active treatment with age- and sex-matched healthy controls. The primary objective was to assess differences in HRQoL and work productivity between groups and explore clinical, endoscopic, and occupational factors associated with impaired outcomes. All participants underwent a single, structured in-person evaluation using validated questionnaires and standardized clinical assessments.

A total of 371 consecutive patients with IBD were recruited over a prespecified four-year period (October 2018 to October 2024) from two tertiary referral centers in Northern Greece: University General Hospital of Alexandroupolis (n = 169) and Theageneio General Hospital of Thessaloniki (n = 202). Inclusion criteria comprised a confirmed diagnosis of CD or UC, ongoing pharmacological treatment at the time of recruitment, and age between 15 and 65 years. Patients not meeting these criteria or those who declined participation were excluded from the study.

For each patient, demographic, clinical, and endoscopic data were collected. Variables included age, gender, occupation, disease duration (years since initial diagnosis), current treatment regimen, and disease location classified according to the Montreal Classification [12]. Disease activity was assessed using the Harvey-Bradshaw Index (HBI) for CD [13] and the Partial Mayo Score (PMS) for UC [14]. Endoscopic activity was evaluated using the Simple Endoscopic Score for Crohn’s Disease (SES-CD) in patients with CD [15], the Rutgeerts score in patients with CD previously surgically treated for the disease [16], and the Mayo Endoscopic Score (MES) in patients with UC [17]. Additional clinical parameters included the presence of perianal disease (in CD) and the presence of EIMs (e.g., arthritis, dermatologic or ocular involvement).

Control group

An equal number of age- and sex-matched healthy controls (n = 371) were recruited during the same study period. The control group was selected from the general population associated with the participating hospitals, including community volunteers and hospital staff. All control participants underwent standardized screening, including a structured medical history, to confirm eligibility. Participants aged 15-65 years without a history of IBD or other chronic inflammatory disorders were eligible for inclusion. Healthy controls completed the same validated questionnaires and standardized clinical assessments as the patient cohort.

Patient-reported outcome measures

Quality of life and work productivity were assessed using two validated questionnaires. The EQ-5D-3L is a standardized instrument for assessing HRQoL. It includes a descriptive system covering five domains, namely, mobility, self-care, usual activities, pain/discomfort, and anxiety/depression, each rated on a three-point scale (1 = no problems, 2 = some problems, 3 = extreme problems). In addition, the EQ Visual Analogue Scale (EQ VAS) records respondents’ self-rated health on a 0-100 scale (health state), where 0 represents the worst imaginable health and 100 the best imaginable health, providing a simple, subjective summary of perceived health [5]. The WPAI is a validated instrument for assessing the impact of health problems on work and daily activities. It measures four domains, namely, absenteeism (work time missed), presenteeism (days when the amount or type of work performed was limited, when less was accomplished than intended, or when tasks were completed with reduced accuracy or care), overall work productivity loss (a combination of absenteeism and presenteeism), and activity impairment (periods when daily activities were limited or when less was accomplished than intended). Outcomes are expressed as percentages, with higher values indicating greater impairment [6,18].

Statistical analysis

For normally distributed continuous variables, means and standard deviations (SDs) were used for descriptive statistics, and the t-test was implemented for comparison between patients and controls. In case that normality was not met based on Kolmogorov-Smirnov and Shapiro-Wilk tests, medians and interquartile ranges (IQRs) were alternatively utilized, while comparisons were performed using the independent-samples Mann-Whitney U test. Proportions were compared using the chi-square test. Multivariable models included independent variables that differed between groups and were built using categorical regression with optimal scaling. The latter was performed after maximal discretization (up to seven categories in the case of continuous variables), ridge regression, and 10-fold cross-validation using the CATREG SPSS algorithm. Multicollinearity issues were minimized by setting the maximum tolerance allowed to 0.5. The level of statistical significance was set to a p-value <0.05. All numerical values were expressed with at least two significant digits. The SPSS version 26.0.0.0 software (IBM Corp., Armonk, NY, USA) was used for statistical analysis.

Ethical considerations

All clinical and endoscopic indices used in this study (HBI, PMS, SES-CD, MES, and Rutgeerts score) are validated, widely accepted tools for the assessment of IBD activity and do not require special permission for use in academic or clinical research settings. Written permission for the use of the WPAI instrument was not required, nor was it obtained; instead, appropriate citation of the original publications was ensured in accordance with ethical and scientific standards. The EQ-5D instrument was utilized following registration approval on the EuroQol website (registration number: 78701, Democritus University of Thrace), with proper acknowledgment and citation of the original sources.

The study was conducted in accordance with the regulations of the Institutional Review Board and the Ethics and Scientific Committee of the University General Hospital of Alexandroupolis (approval number: 941/11-10-2018). All participants provided written informed consent before inclusion. The study was conducted in accordance with the principles of the Declaration of Helsinki (2013).

## Results

Study population

A total of 371 patients with IBD and 371 healthy controls were included in the analysis. The IBD cohort comprised 63.9% males (n = 237) and 36.1% females (n = 134), while the control group included 62.8% males (n = 233) and 37.2% females (n = 138). The mean age did not differ significantly between groups (42.4 ± 14.2 vs. 43.1 ± 13.7 years, p > 0.05). Regarding employment, 66.6% of patients with IBD were actively employed compared with 86.5% of controls, while 33.4% of patients and 13.5% of controls were not working. Notably, 14.8% of IBD patients reported having discontinued employment specifically because of their disease. Among patients with IBD, 59.8% were diagnosed with CD and 40.2% with UC. The mean age at diagnosis was 34.3 ± 13.3 years, and the mean disease duration was 8.1 ± 6.7 years (Table 1).

**Table 1:** Demographics and clinical characteristics of the IBD cohort. *: Chi-square test (df = 1); **: Independent-samples Mann-Whitney U test (standardized). Data are presented as N (%) or median (IQR), as the Lilliefors and/or Kolmogorov-Smirnov test indicated non-normality in at least one group. Group comparisons were performed using the Mann-Whitney U or chi-square test, as appropriate. A p-value <0.05 was considered statistically significant. Note: ^a^: Disease phenotype of Crohn’s disease was classified using Montreal classification: age at diagnosis: A1 (<17 years), A2 (17–40 years), A3 (>40 years); L1, ileal; L2, colonic; L3, ileocolonic; B1, no stricturing; B2, stricturing; B3, penetrating. ^b^: Disease location was classified using Montreal classification: E1, proctitis; E2, left-sided colitis; E3, pancolitis. AZA: azathioprine; CD: Crohn’s disease; IBD: inflammatory bowel disease; IQR: interquartile range; NA: not available; MES: Mayo Endoscopic Score; SD: standard deviation; SES-CD: Simple Endoscopic Score for Crohn’s disease; UC: ulcerative colitis

Characteristics	Total (N = 371)	UC (N = 149)	CD (N = 222)	P-value	Statistic
Demographics					
Male gender, N (%)	237 (63.9)	95 (63.8)	142 (64)	0.968	0.002*
Mean age at inclusion, years, median (IQR)	42.0 (23.0)	46.0 (23.0)	41.0 (22.0)	0.010	2.590**
Mean age at diagnosis, years, median (IQR)	33.0 (22.0)	35.0 (21.5)	32.0 (20.0)	0.009	2.606**
Mean disease duration, years, median (IQR)	6.0 (8.0)	6.0 (7.5)	6.0 (7.3)	0.589	0.541**
Disease phenotype of CD^a^ (%)					
A1/A2/A3	NA	NA	9.5%/58.1%/32.4%	NA	NA
L1/L2/L3	NA	NA	45.5%/15.8%/38.7%	NA	NA
B1/B2/B3	NA	NA	59.5%/22.1%/18.5%	NA	NA
Disease phenotype of UC^b^ (%)					
E1/E2/E3	NA	24.2%/45%/30.9%	NA	NA	NA
Clinical characteristics					
Extraintestinal manifestation, N (%)	70	12	58	<0.001	19.02*
Perianal or fistulizing disease, N (%)	NA	NA	24	NA	NA
Surgery, N (%)	68	5	63	<0.001	37.293*
Clinical score at inclusion, median (IQR)					
HBI	NA	NA	2 (0–11)	NA	NA
Partial Mayo	NA	2 (0–7)	NA	NA	NA
Biomarkers, median (IQR)					
Hemoglobin, g/L, median (IQR)	12.6 (1.9)	12.7 (1.9)	12.3 (1.8)	<0.001	3.839**
Platelets, ×10^9^/L, median (IQR)	288 (50)	283 (36)	289 (73)	0.164	1.391**
WBC, ×10^9^/L, median (IQR)	7.9 (2.7)	8.3 (2.8)	7.8 (2.8)	0.016	2.401**
C-reactive protein, mg/dL, median (IQR)	1.6 (2.4)	1.5 (2.5)	1.7 (2.3)	0.105	1.622**
Endoscopic score, median (IQR)					
SES-CD	NA	NA	3 (1–8)	NA	NA
Rutgeerts	NA	NA	2 (1–4)	NA	NA
MES	NA	2 (0–7)	NA	NA	NA
Treatment at inclusion, N (%)					
Aminosalicylates	153 (41.2)	122 (81.9)	31 (14)	<0.001	169.71*
Corticosteroids	38 (10.2)	19 (12.8)	19 (8.6)	0.192	1.705*
Immunomodulators	58 (15.6)	18 (12.1)	40 (18)	0.123	2.383*
Biologics	223 (60.1)	47 (21.2)	176 (79.3)	<0.001	84.729*
Concomitant biologics and AZA	36 (9.7)	10 (6.7)	26 (11.7)	0.111	2.544*

The mean health state (0-100) was 78.0 ± 15.3; median inflammatory markers were low (C-reactive protein: 2.24 ± 2.22 mg/L; white blood cell count: 7,854 ± 1,867/μL). Overall, 60.1% of patients were receiving biologic therapy. Prior surgery had been performed in 18.3% of IBD patients, who reported a lower self-reported health state (73.2 ± 17.0 vs. 79.0 ± 15.0, p = 0.004). EIMs were present in 18.9% and were associated with greater impairment in usual activities and mobility, higher disease activity, and worse work/activity impairment (all p < 0.001). Overall health state was also lower among patients with EIMs (71.7 ± 16.8 vs. 79.4 ± 14.5, p < 0.001). Arthritis represented the most prominent EIM (p < 0.001).

Health-related quality of life (EQ-5D) and work productivity and activity impairment (WPAI)

Across all dimensions of the EQ-5D-3L, patients with IBD reported significantly greater impairment than healthy controls. Median scores were higher for mobility (p = 0.001), self-care (p = 0.002), usual activities (p < 0.001), pain/discomfort (p < 0.001), and anxiety/depression (p < 0.001). Similarly, work productivity and activity were significantly impaired in the IBD group compared with healthy controls, as assessed by the WPAI questionnaire. Patients with IBD reported higher absenteeism (p = 0.004), presenteeism (p < 0.001), overall work productivity loss (p < 0.001), and activity impairment (p < 0.001) (Tables 2, 3).

**Table 2:** Descriptive statistics and group comparability. *: Chi-square test (df=1); **: Independent-samples Mann-Whitney U test (standardized). Data are presented as median (IQR), as the Lilliefors and/or Kolmogorov-Smirnov tests indicated non-normality in at least one group. Gender is represented as N. Continuous variables were compared between groups using the Mann–Whitney U test and categorical variables using the chi-square test. A p-value <0.05 was considered statistically significant. EQ-5D-3L: EuroQol 5-Dimension, 3-Level Version; IQR: interquartile range; WPAI: Work Productivity and Activity Impairment Questionnaire

Characteristics	Patients (N = 371)	Controls (N = 371)	Statistic	P-value
Gender (males/females)	237/134	133/138	0.093*	0.761
Age, years, median (IQR)	40 (16)	42 (20)	0.583**	0.560
Health state, median (IQR)	80 (20)	100 (10)	14.914**	<0.001
EQ-5D-3L, median (IQR)				
Mobility	1 (0)	1 (0)	3.392**	0.001
Self-care	1 (0)	1 (0)	3.128**	0.002
Usual activities	1 (0)	1 (0)	4.902**	<0.001
Pain/Discomfort	1 (1)	1 (0)	7.816**	<0.001
Anxiety/Depression	2 (1)	1 (0)	8.575**	<0.001
WPAI, median (IQR)				
Absenteeism (%)	0 (0)	0 (0)	2.872**	0.004
Presenteeism (%)	10 (30)	0 (0)	14.604**	<0.001
Activity impairment (%)	10 (20)	0 (0)	15.511**	<0.001
Work productivity loss (%)	10 (30)	0 (0)	12.361**	<0.001

**Table 3:** Descriptive statistics and group comparisons of HRQoL measures between patients and controls. *: Chi-square test (df = 2); **: Independent-samples Mann-Whitney U test (standardized); *** Categorical regression analysis of variance F (df = 1). Data are presented as N (%) or median (IQR), as the Lilliefors and/or Kolmogorov-Smirnov test indicated non-normality in at least one group. Continuous variables were compared between groups using the Mann–Whitney U test and categorical variables using the chi-square test. Additionally, categorical regression was applied to analyze the EQ-5D-3L components. A p-value <0.05 was considered statistically significant. EQ-5D-3L: EuroQol 5-Dimension, 3-Level Version; HRQoL: health-related quality of life; IQR: interquartile range; SD: standard deviation; UC: ulcerative colitis; WPAI: Work Productivity and Activity Impairment Questionnaire

Characteristics	Patients (N = 371)	Controls (N = 371)	Statistic	P-value
Male gender, N (%)	237 (63.9)	233 (62.8)	0.093*	0.761
Age at inclusion, years, median (IQR)	42.0 (23.0)	43.0 (22.0)	0.583**	0.560
Active occupation, N (%)	247 (66.6)	321 (86.5)	41.112*	<0.001
EQ-5D-3L				
Mobility	-	-	12.276* 15.514***	0.002 0.002
1, N (%)	315 (84.9)	344 (92.7)		
2, N (%)	54 (14.6)	27 (7.3)		
3, N (%)	2 (0.5)	0 (0)		
Self-care			10.040* 7.587 ***	0.006 0.006
1, N (%)	342 (92.2)	361 (97.3)		
2, N (%)	28 (7.5)	10 (2.7)		
3, N (%)	1 (0.3)	0 (0)		
Usual activities			25.174* 19.321***	<0.001 <0.001
1, N (%)	286 (77.1)	335 (90.3)		
2, N (%)	81 (21.8)	36 (9.7)		
3, N (%)	4 (1.1)	0 (0)		
Pain/Discomfort			61.180* 73.824***	<0.001 <0.001
1, N (%)	229 (61.7)	322 (86.8)		
2, N (%)	137 (36.9)	48 (12.9)		
3, N (%)	5 (1.3)	1 (0.3)		
Anxiety/Depression			74.468* 55.851***	<0.001 <0.001
1, N (%)	173 (46.6)	287 (77.4)		
2, N (%)	176 (47.4)	76 (20.5)		
3, N (%)	22 (5.9)	8 (2.2)		
Health State, 1–100, median (IQR)	80 (20–100)	100 (40–100)	14.914**	<0.001

**Figure 1:**
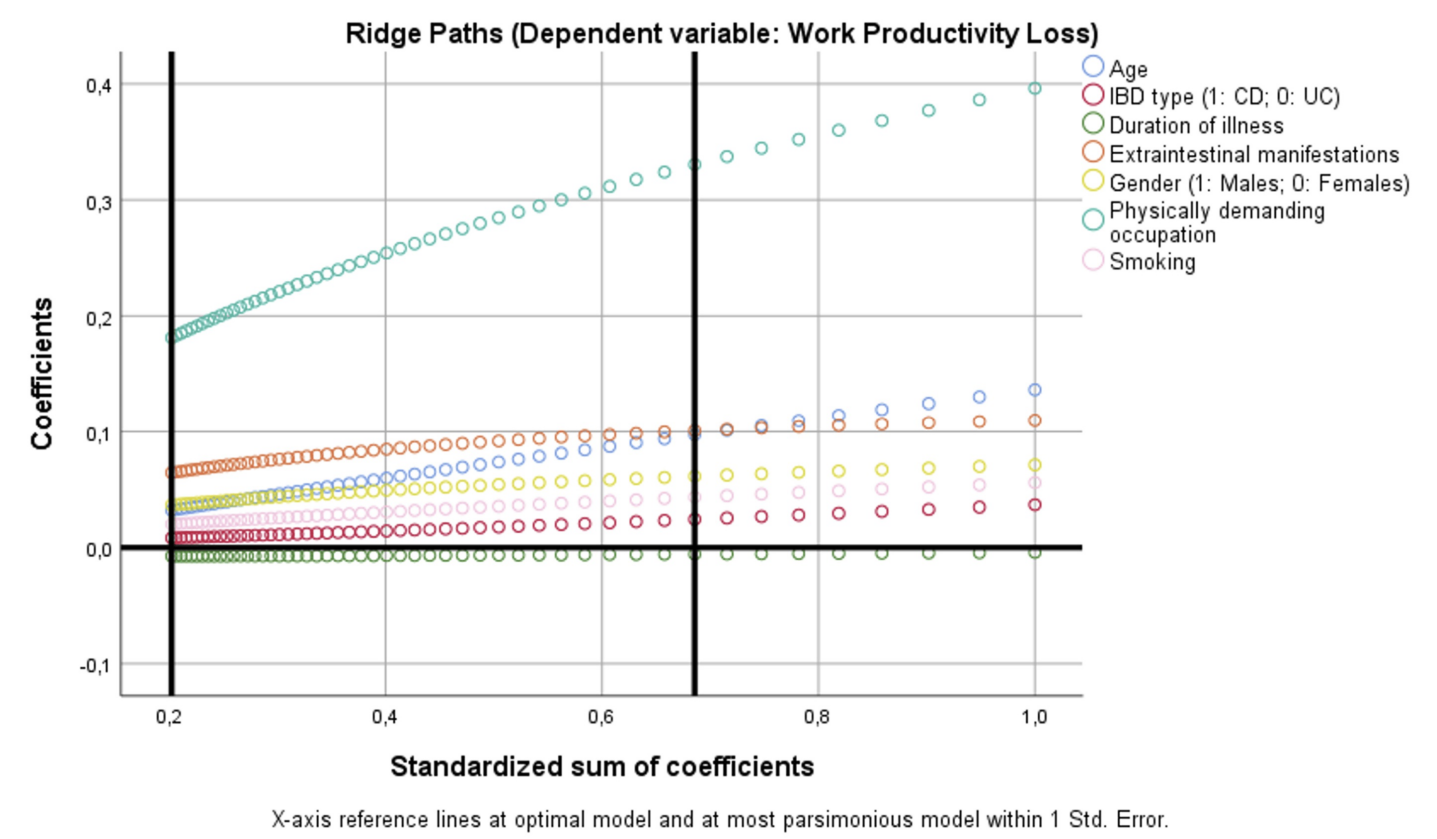
Binary regression model based on optimal scaling after discretization into the maximum of categories allowed (binary = 2; scale = 7), ridge regression, and 10× cross-validation assessing the parameters that are independently correlated with work productivity loss. CD: Crohn’s disease; IBD: inflammatory bowel disease; UC: ulcerative colitis

For work productivity loss, a physically demanding occupation emerged as the strongest (p < 0.001). EIMs were also independently associated (p = 0.033), whereas no associations were found with IBD type (p = 0.641), disease duration (p = 0.783), gender (p = 0.163), age (p = 0.308), or smoking (p = 0.317) (Table 4) (Figure 1).

**Table 4:** Categorical regression multivariate full model used to assess the impact of disease type, duration of illness, gender, age, smoking, occupation type, and extraintestinal manifestations as potential prognosticators of work productivity loss. Differences between groups were analyzed using analysis of variance (F-value and p-value shown). Regularized R^2^ = 0.117; analysis of variance p < 0.001. CD: Crohn’s disease; UC: ulcerative colitis

Parameter	Quantification factor	Standardized beta	Standardized beta error^†^	F	Tolerance^‡^	P-value
Disease type		0.008	0.018	0.218	0.884	0.641
*UC*	-1.173					
*CD*	0.853					
Disease duration, years		-0.007	0.027	0.076	0.820	0.783
1–2	-1.225					
*3–6*	-0.494					
*7–9*	0.237					
*10–13*	0.968					
*14–18*	1.699					
*19–37*	2.430					
Gender		0.037	0.026	1.958	0.963	0.163
*Females*	1.574					
*Males*	-0.635					
Age, years		0.032	0.031	1.046	0.766	0.308
*15–19*	-2.060					
*20–29*	-1.319					
*30–38*	-0.579					
*39–46*	0.162					
*47–54*	0.903					
*55–65*	1.643					
Smoking		0.020	0.020	1.007	0.934	0.317
*No*	0.541					
*Yes*	-1.847					
Occupation type		0.181	0.026	47.151	0.862	<0.001
Office	-0.918					
Physically demanding	1.089					
Extraintestinal manifestations		0.065	0.030	4.620	0.843	0.033
No	-0.446					
Yes	2.242					

**Figure 2:**
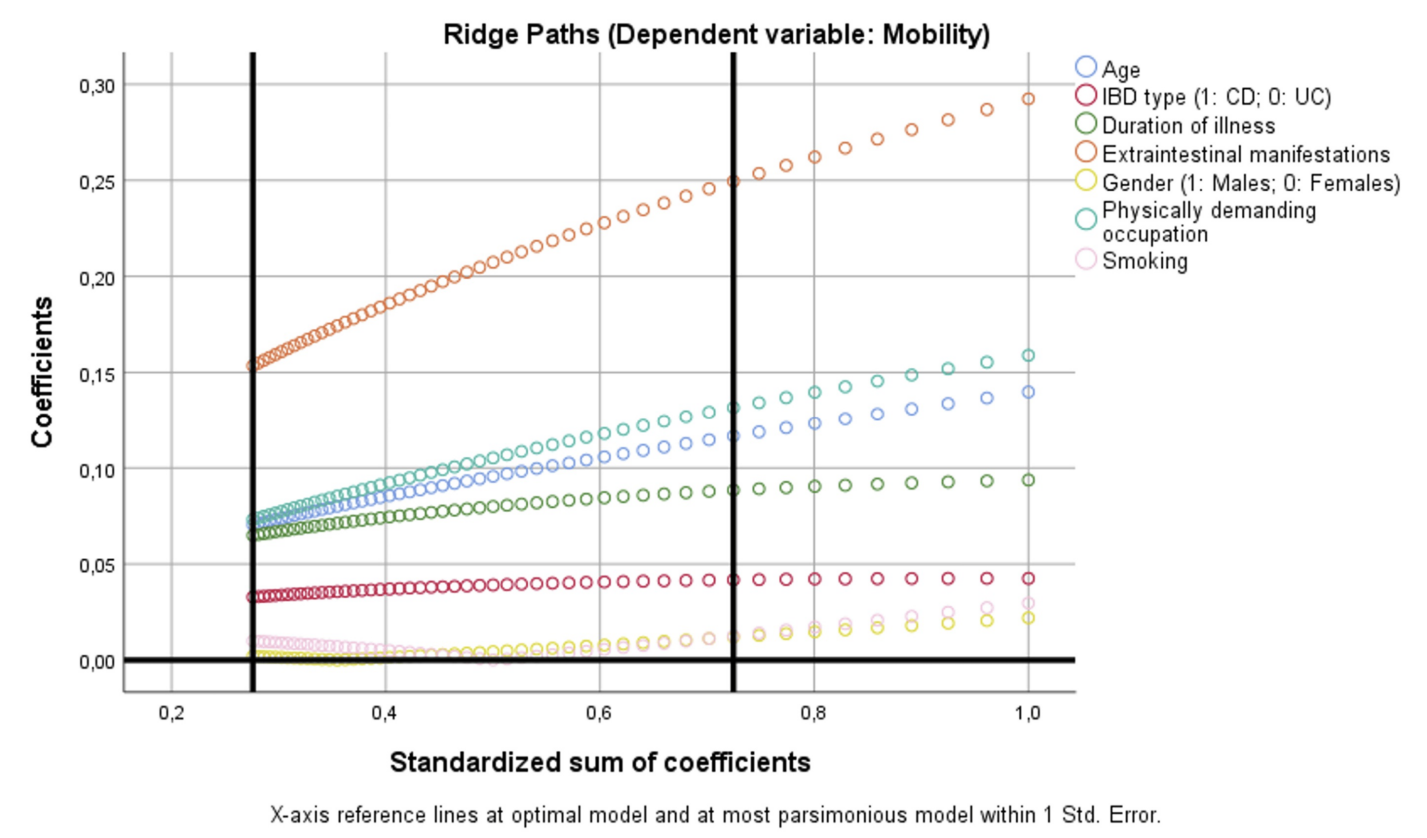
Binary regression model based on optimal scaling after discretization into the maximum of categories allowed (binary = 2; scale = 7), ridge regression, and 10× cross-validation assessing the parameters that are independently correlated with mobility. CD: Crohn’s disease; IBD: inflammatory bowel disease; UC: ulcerative colitis

Similarly, regarding mobility, disease duration (p = 0.015), age (p < 0.001), physically demanding occupation (p = 0.003), and EIMs (p < 0.001) were significant predictors. Ridge paths confirmed that age and disease duration demonstrated steadily increasing coefficients, highlighting their robust contribution to mobility impairment. On the contrary, IBD type, gender, and smoking showed negligible effects (Figure 2).

Self-care limitations were independently related to smoking (p = 0.004), physically demanding occupation (p = 0.006), and EIMs (p < 0.001). Ridge regression paths indicated that the effect of EIMs was particularly stable, whereas IBD type, disease duration, gender, and age demonstrated near-zero coefficients throughout penalization levels.

**Figure 3:**
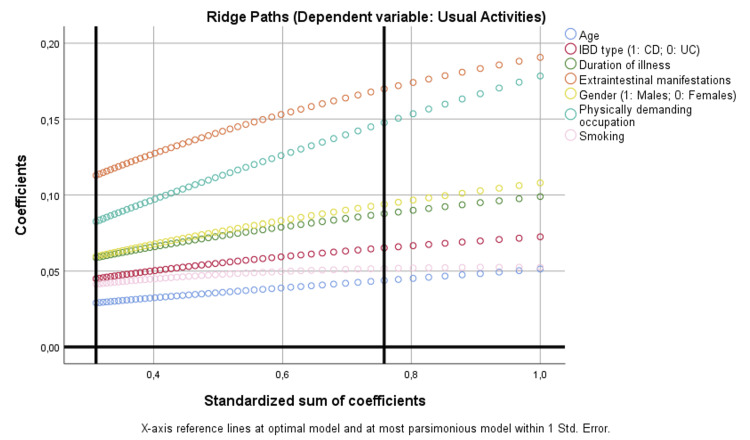
Binary regression model based on optimal scaling after discretization into the maximum of categories allowed (binary = 2; scale = 7), ridge regression, and 10× cross-validation assessing the parameters that are independently correlated with usual activities. CD: Crohn’s disease; IBD: inflammatory bowel disease; UC: ulcerative colitis

For usual activities, CD (p = 0.007), longer disease duration (p = 0.006), male gender (p = 0.005), physically demanding occupation (p = 0.001), and EIMs (p < 0.001) were all significant determinants. Ridge paths highlighted IBD type and gender as increasingly important predictors under more parsimonious models, while age and smoking remained negligible (Figure 3).

**Figure 4:**
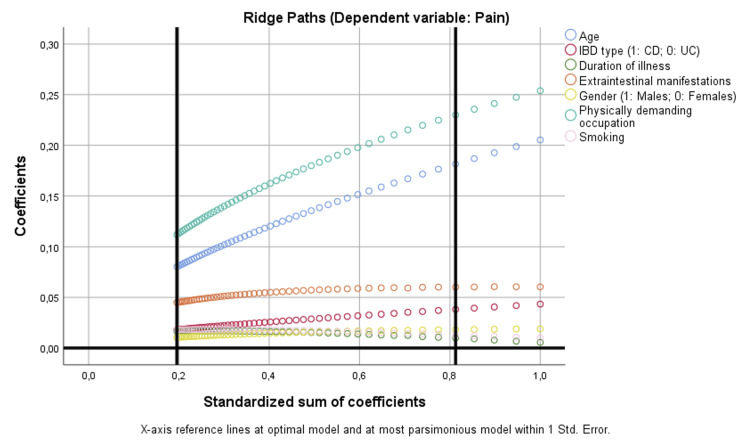
Binary regression model based on optimal scaling after discretization into the maximum of categories allowed (binary = 2; scale = 7), ridge regression, and 10× cross-validation assessing the parameters that are independently correlated with pain. CD: Crohn’s disease; IBD: inflammatory bowel disease; UC: ulcerative colitis

In the pain/discomfort domain, age (p = 0.002) and physically demanding occupation (p < 0.001) were independent predictors, consistent with ridge paths where these variables demonstrated strong and stable positive coefficients. Other factors, including IBD type, disease duration, gender, smoking, and EIMs, had little explanatory contribution (Figure 4).

**Figure 5:**
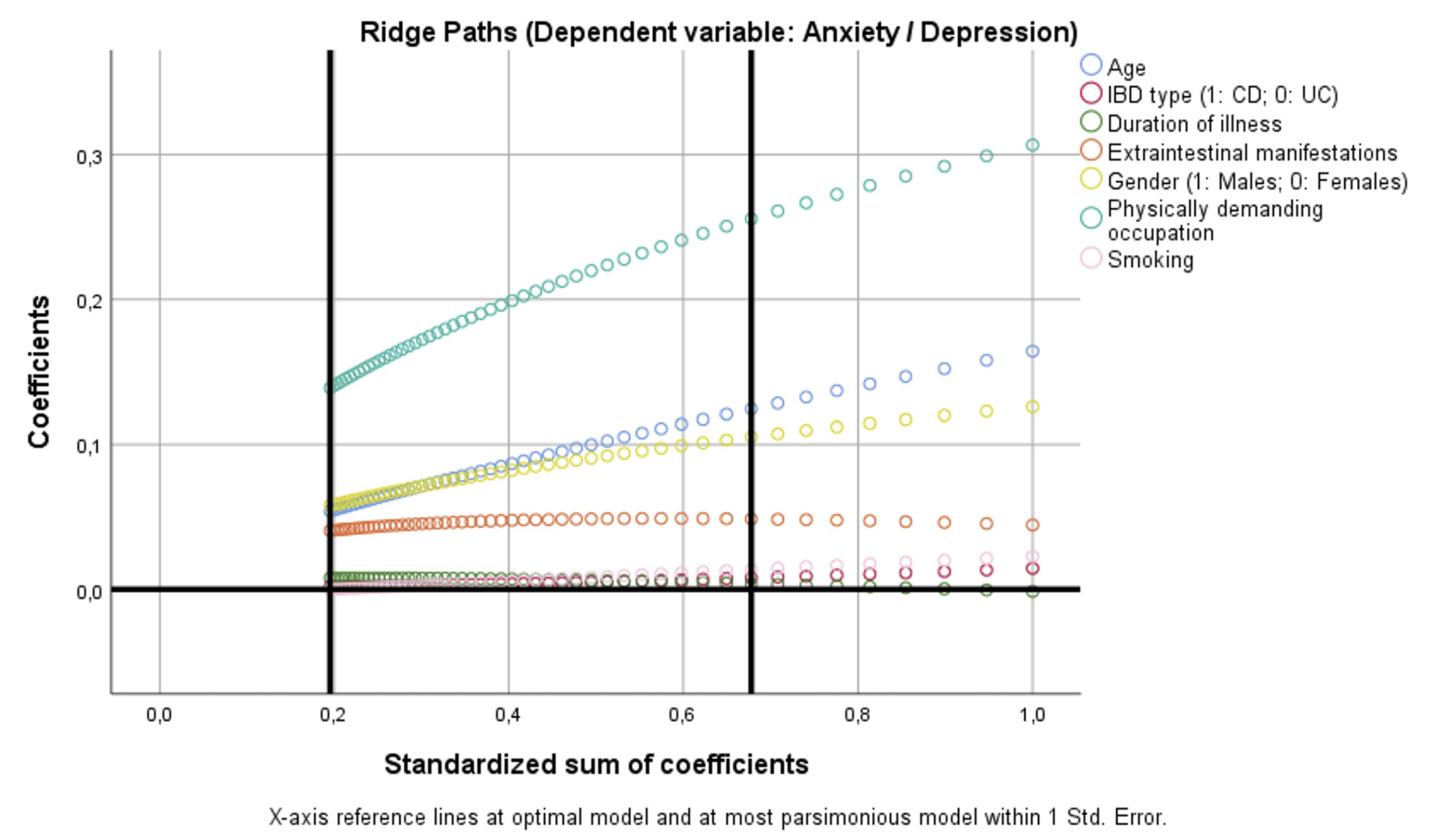
Binary regression model based on optimal scaling after discretization into the maximum of categories allowed (binary = 2; scale = 7), ridge regression, and 10× cross-validation assessing the parameters that are independently correlated with anxiety/depression. CD: Crohn’s disease; IBD: inflammatory bowel disease; UC: ulcerative colitis

For anxiety/depression, female gender (p = 0.033) and physically demanding occupation (p < 0.001) were independently associated. Ridge paths further emphasized physically demanding occupation as the most influential determinant across penalization levels, while the contribution of gender remained stable but smaller in magnitude. No significant associations were observed for IBD type, disease duration, age, smoking, or EIMs (Figure 5).

Endoscopic disease activity correlated significantly with HRQoL and work productivity outcomes. In UC, higher MES was associated with lower EQ-VAS health state (p < 0.001) and greater absenteeism (p = 0.001), presenteeism (p < 0.001), overall work productivity loss (p < 0.001), and activity impairment (p < 0.001). MES also showed significant associations with EQ-5D-3L domains, including usual activities (p < 0.001), pain/discomfort (p < 0.001), anxiety/depression (p < 0.001), and, more modestly, self-care (p = 0.023). Similarly, in CD, higher SES-CD values correlated with worse EQ-VAS (p < 0.001) and increased absenteeism (p = 0.010), presenteeism (p = 0.011), productivity loss (p = 0.009), and activity impairment (p = 0.004). SES-CD was further related to impaired mobility (p < 0.001), usual activities (p = 0.001), pain/discomfort (p < 0.001), and anxiety/depression (p = 0.008). In contrast, Rutgeerts scores did not demonstrate significant correlations with either HRQoL or work productivity measures.

In addition, both the HBI and the PMS were strongly correlated with overall health outcomes. Specifically, both scores correlated negatively with health state (HBI: rho = -0.352, PMS: rho = -0.382; both p < 0.0001) and health impairment (HBI: rho = -0.344, PMS: rho = -0.410; both p < 0.0001).

**Figure 6:**
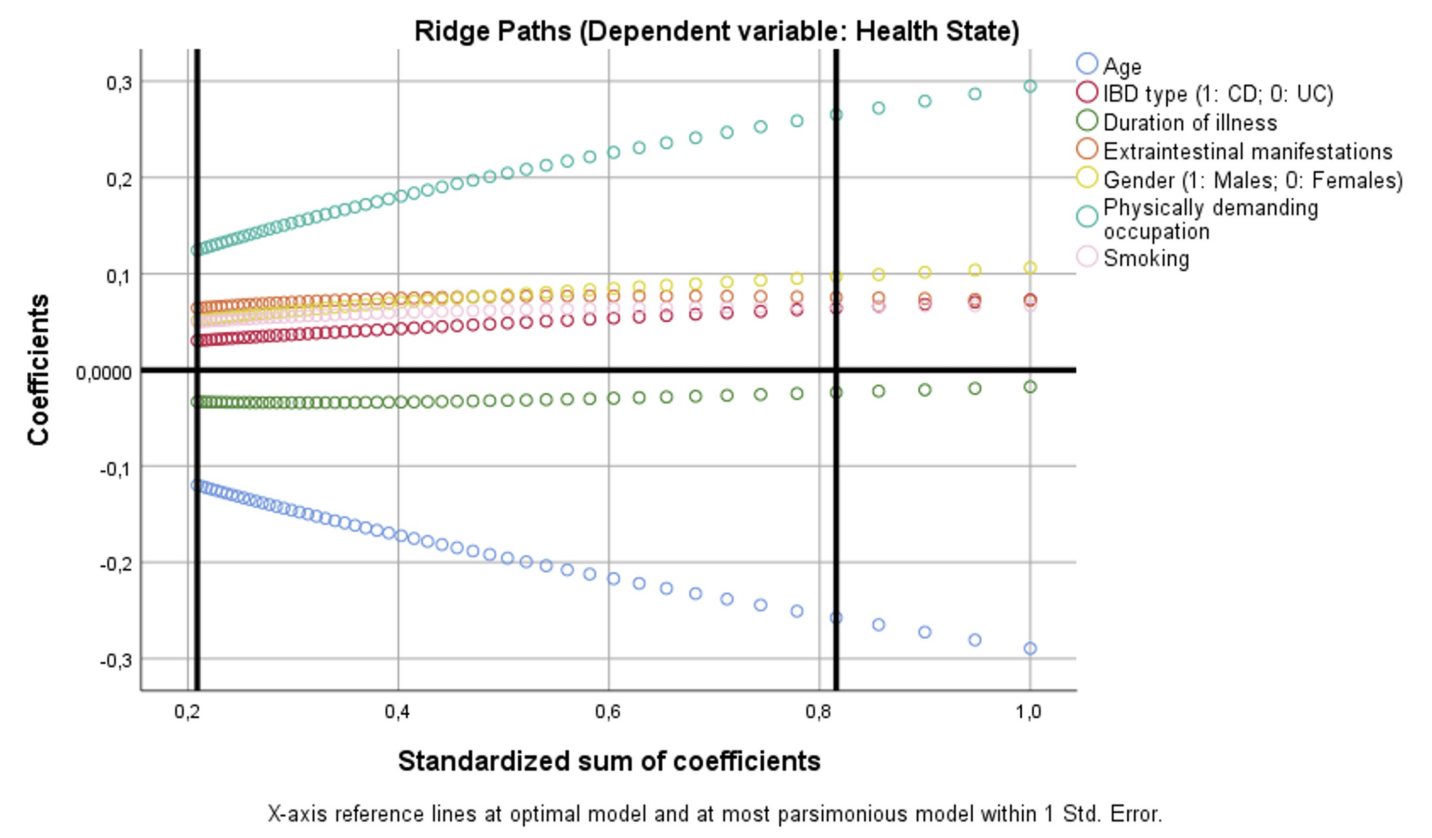
Binary regression model based on optimal scaling after discretization into the maximum of categories allowed (binary = 2; scale = 7), ridge regression, and 10× cross-validation assessing the parameters that are independently correlated with health state. CD: Crohn’s disease; IBD: inflammatory bowel disease; UC: ulcerative colitis

**Figure 7:**
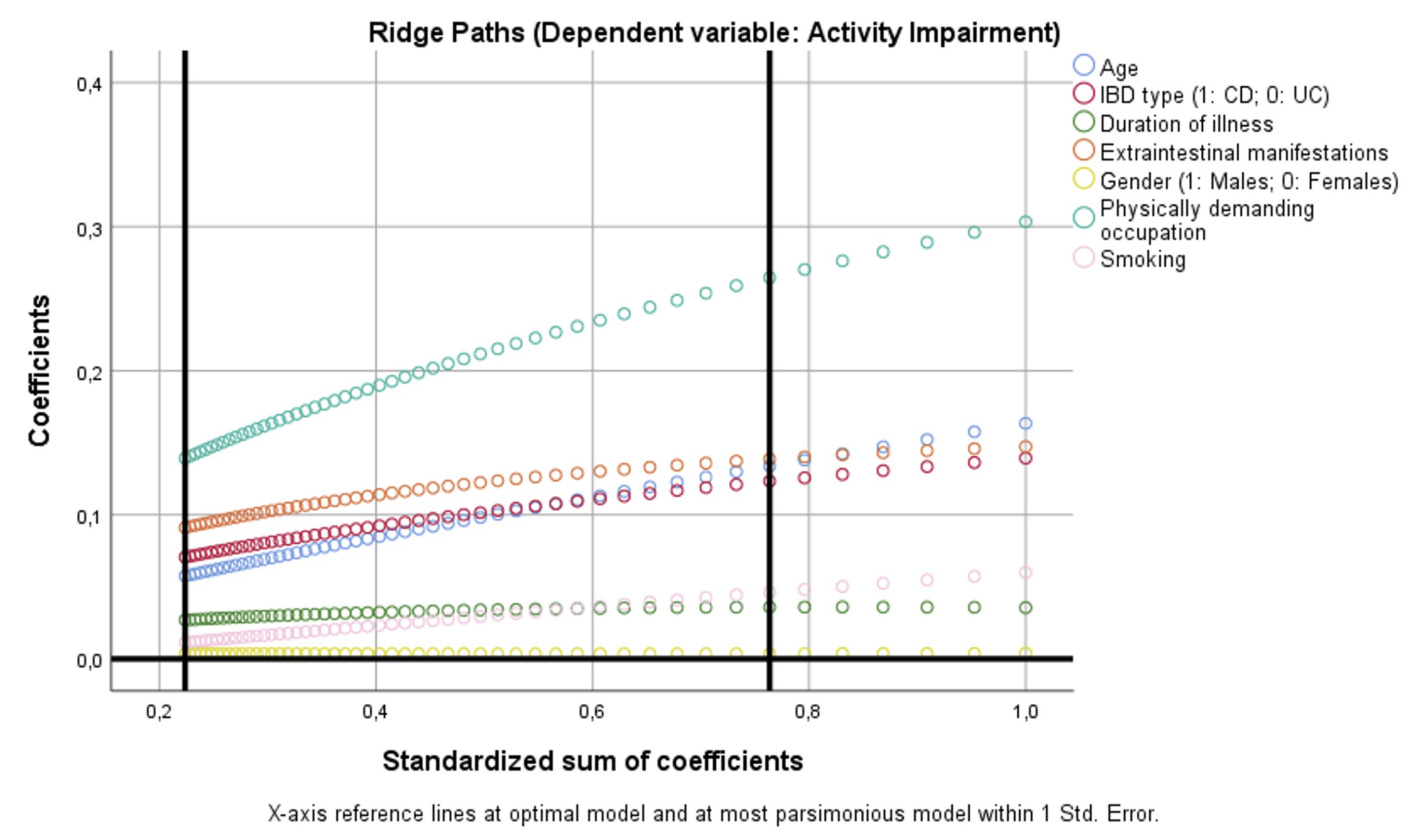
Binary regression model based on optimal scaling after discretization into the maximum of categories allowed (binary = 2; scale = 7), ridge regression, and 10× cross-validation assessing the parameters that are independently correlated with activity impairment. CD: Crohn’s disease; IBD: inflammatory bowel disease; UC: ulcerative colitis

Finally, ridge path visualizations further supported these associations, indicating that age and physically demanding occupation were among the most influential covariates in health state and activity impairment models (Figures 6, 7).

## Discussion

Recent studies reinforce the central role of multidimensional patient-reported outcomes in IBD, validating robust instruments, standardizing outcome sets, and embedding patient-centered measures into routine care and trials to guide targeted management and a holistic approach [7-9]. This study demonstrates that patients with IBD experience significantly greater impairment in HRQoL and work productivity compared with age- and gender-matched healthy controls, despite ongoing treatment. The greatest impairments were recorded in the domains of pain/discomfort and anxiety/depression, underscoring the combined physical and psychological burden of IBD, even in treated patients [3,4,19] (Table 3). Furthermore, IBD patients reported markedly higher absenteeism, presenteeism, and overall productivity loss, confirming earlier findings that the occupational and functional burden of IBD extends well beyond the clinical manifestations of the disease [6,10,11,20].

**Figure 8:**
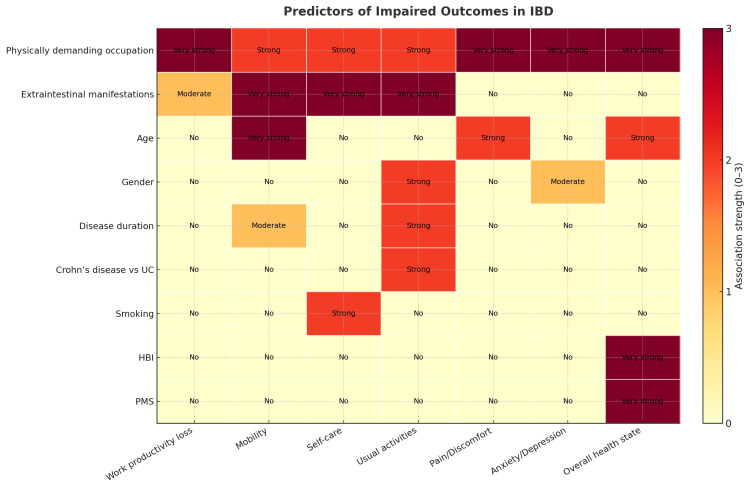
Predictors of impaired outcomes in IBD. Heatmap depicting the strength of association between clinical predictors and patient-reported outcomes. Colors and in-cell labels indicate association strength on a numeric scale: 0 = No, 1 = Moderate (0.01 ≤ p < 0.05), 2 = Strong (0.001 ≤ p < 0.01), 3 = Very strong (p < 0.001). Associations derive from multivariable models supported by ridge-regression paths; “Gender” is shown as a unified predictor. HBI: Harvey-Bradshaw Index; IBD: inflammatory bowel disease; PMS: Partial Mayo Score; UC: ulcerative colitis

This approach identified physically demanding occupations as the most consistent determinant of impaired HRQoL and work productivity. This finding is clinically relevant, as it suggests that the interaction between disease burden and occupational strain contributes significantly to patients’ functional limitations. Previous studies have similarly reported that physically demanding work exacerbates the impact of chronic illness, including IBD, by amplifying fatigue, musculoskeletal strain, and absenteeism [6,21]. Our results extend this knowledge by demonstrating that these effects are consistent across multiple health domains, even after penalization of less influential covariates. EIMs also emerged as important predictors of impairment, particularly in mobility, self-care, and usual activities (Figure 8).

This aligns with earlier work showing that EIMs, such as arthritis, dermatologic conditions, and ocular involvement, are not only frequent but also strongly associated with diminished HRQoL [2,22]. Their contribution to disability appears independent of intestinal disease activity, highlighting the importance of systematic screening and management of EIMs in routine clinical practice.

Interestingly, the association between endoscopic findings and patient-reported outcomes highlights the strong relationship between objective mucosal inflammation and the subjective burden of disease in IBD. Both MES and SES-CD were consistently associated with impaired quality of life and reduced work productivity, underlining that mucosal activity is not only a prognostic factor but also translates into tangible functional and psychosocial consequences for patients. The lack of association observed with the Rutgeerts score may reflect the smaller sample size or the distinct postoperative setting, suggesting that different indices capture different aspects of disease burden. These results reinforce the importance of targeting endoscopic remission to optimize not only clinical outcomes but also patient-reported well-being and productivity.

Furthermore, disease activity scores (HBI and PMS) were strongly correlated with overall health state and activity impairment, but other factors, such as IBD type or smoking status, contributed minimally across most domains (Figure 8). The notable exception was CD, which was independently associated with greater impairment in usual activities, reflecting the typically more aggressive and disabling course of CD relative to UC [23]. Age and disease duration were additional contributors, particularly to mobility and pain, findings that likely reflect the cumulative effect of chronic inflammation, structural damage, and comorbidities over time [24]. The consistent negative correlations between disease activity indexes (HBI and PMS) and both health state and health impairment further underscore the central role of disease control in optimizing patient outcomes. These results align with recent treat-to-target recommendations emphasizing not only endoscopic remission but also improvements in HRQoL as essential therapeutic goals [25].

Gender differences were also domain-specific, with female gender associated with higher levels of pain and anxiety/depression, while male gender was linked to worse impairment in usual activities. Prior studies have documented greater pain perception and psychological burden among women with IBD [26], while men may experience more restrictions in physical or occupational functioning, which may explain these divergent findings.

To our knowledge, the retrospective study by Wetwittayakhlang et al. is the only investigation directly comparable to ours in scope, instruments, and clinical endpoints. They retrospectively studied 112 IBD patients and found that patient-reported outcomes correlated strongly with clinical remission but only moderately with biomarkers and endoscopic remission [27]. Our larger study emphasized broader patient-reported outcome domains (EQ-5D-3L, WPAI). While not longitudinal, we observed HBI/PMS associations with poorer health state and identified EIMs and physically demanding occupation as independent drivers of reduced quality of life and productivity. Thus, our data complement prior work by broadening the patient-reported outcome focus in a larger, controlled cohort.

Recent evidence highlights the increasing use and importance of patient-reported outcomes in the monitoring of IBD. Horrigan et al. demonstrated that while multiple validated patient-reported outcome instruments exist for IBD, their routine integration into clinical practice remains limited, emphasizing the need for standardized implementation [28]. In this context, our study contributes to bridging this gap by supporting the practical use of patient-reported outcomes as a clinical tool in IBD management. In a systematic review and meta-analysis, Youssef et al. reported substantial work productivity impairment among IBD patients, with pooled estimates of absenteeism, presenteeism, and overall work impairment reaching 16.4%, 35.9%, and 39.4%, respectively, underscoring the significant occupational burden of the disease [29]. Consistent with these findings, our study also demonstrated significant impairment in both work productivity and quality of life, further emphasizing the impact of IBD on patients’ functional and psychosocial well-being. Similarly, Hiraoka et al. found that despite active treatment, patients with UC continued to experience reduced HRQoL and marked work productivity loss [30]. In agreement with these results, our study also demonstrated that patients receiving ongoing treatment showed no significant improvement in work productivity or quality of life, highlighting this persistent impairment as one of the major findings of the study. Together, these findings reinforce the value of patient-reported outcomes as essential tools for comprehensive IBD surveillance and management.

The present study has several strengths. It includes a large, well-characterized cohort with matched controls, validated and widely used instruments (EQ-5D-3L, WPAI), and penalized regression modeling, mitigating major sources of bias [5,6]. However, this study has certain limitations. Its cross-sectional design precludes causal inference, and residual confounding from unmeasured psychosocial or economic factors cannot be entirely excluded. The absence of longitudinal follow-up to capture temporal variability further limits the interpretation of changes over time. Moreover, as the study population was recruited from tertiary centers, patients with more complex disease may be overrepresented, potentially reducing generalizability.

Finally, clinicians should incorporate structured assessments of occupational status and physical workload when evaluating patients with IBD, as these factors may substantially modify the disease’s functional impact. Multidisciplinary approaches, including occupational counseling, psychological support, and targeted management of EIMs, are essential to address the multidimensional burden of the disease. Interventions tailored to reduce the occupational strain of physically demanding jobs may help preserve work capacity and improve long-term outcomes.

## Conclusions

Patients with IBD under treatment continue to experience significant impairments in HRQoL and work productivity compared with healthy controls. Physically demanding occupation and EIMs were the strongest and most consistent determinants of poor outcomes, while disease activity correlated strongly with health state and activity impairment. These findings highlight the importance of integrated care strategies that extend beyond intestinal inflammation. Comprehensive disease management should incorporate strategies to address pain, psychological distress, and work-related limitations to improve long-term outcomes and quality of life in this patient population.
